# Validation and optimisation of wearable accelerometer data pre-processing for digital measure implementation and development

**DOI:** 10.1371/journal.pone.0346458

**Published:** 2026-10-21

**Authors:** Joss Langford, Jia Ying Chua, Ian Long, Alan C. Williams, Melvyn Hillsdon

**Affiliations:** 1 Department of Public Health and Sport Sciences, University of Exeter, Exeter, United Kingdom; 2 ActivInsights Limited, Kimbolton, Cambridge, United Kingdom; 3 Stoat Group Limited, Devon, United Kingdom; Polytechnic University of Marche: Universita Politecnica delle Marche, ITALY

## Abstract

The increasing use of accelerometers as digital health technologies in clinical trials and clinical care is driving the need for data processing to meet medical standards. The aim of this study was to create and test a modular pipeline for the pre-processing of high-resolution accelerometry that assures the quality, transparency and traceability of digital measures from sensor-level data. The objective is for the pipeline to be a foundational layer in the development, implementation and comparison of measures. The study developed the open GENEAcore package to meet the requirements of regulators, verifying the engineering implementation and analytically validating outputs against reference datasets. Early stages included the optimisation of calibration and non-wear detection. Data-driven detection of behavioural transitions was then validated to give direct bout outputs without the need to identify rules for epoch aggregation and interruptions. The utility for measure development was shown by comparing two algorithms for the characterisation of activity intensity in both the epoch and bout paradigms. Non-wear was detected with a balanced accuracy of 92.3% and the commonly used 13m*g* acceleration standard deviation threshold was empirically validated with a sensitivity analysis for the first time. The detection of transitions proved reliable with 99% detected, on average, within 2 seconds of their occurrence to give a mean expected event duration of 68.6s from a log-normal distribution. The different activity intensity algorithms were more than 99% concordant during movement but their outputs diverged in low movement conditions. Importantly, variable duration bouts created 31% higher daily activity durations compared to 1-second epochs. This evaluation of pre-processing steps has confirmed the attention to detail required to create robust and reproducible results for later clinical validation where small changes in an algorithm or its implementation may have clinically meaningful consequences.

## Introduction

### Overview

Public health and physical behaviour research have been significantly advanced by the advent of wearable raw data accelerometers [1]. Increasingly, these sensor-level measurement systems are used in commercial research to evaluate medical products as digital health technologies [2] and to guide clinical care [3]. In contrast to consumer-grade devices with embedded algorithms, the original measurement outputs are preserved, allowing full verification of the sensor engineering [4] as well as futureproofing for new algorithms.

Wearable sensors make it possible to collect high-resolution information about an individual’s physiology, behaviour and daily routines over long periods with low burden to participants and healthcare professionals. Unlike traditional episodic measures (for example, from clinic visits), these data are gathered continuously in real life. They can be more sensitive, timely, and representative of someone’s complete health and behaviour [5]. These objectively recorded data can eliminate misclassification due to the response biases that come from relying on people’s memory, subjective judgement or self-report [6]. These biases, particularly recall [7] and desirability [8], can lead to over- or under-estimates of time in active behaviours that in turn attenuates associations with health outcomes [9].

However, challenges remain in processing the large volumes of data from sensor-based measurement into digital health measures that are meaningful to both patients and clinicians [10]. To be trusted, digital measures need to be accurate, reliable and clearly associated with health outcomes in the clinical evidence [11]. They also need to be produced through a process that is transparent and repeatable with an understanding of any potential risk to safety [12]. Regulators also insist on full traceability of the intended use throughout the development, implementation and operation of the system [13].

As raw data technologies move from research settings into clinical applications, there is growing pressure to make the way data is handled both more efficient and more trustworthy. Clear and consistent pre-processing, where raw sensor data are prepared before further analysis, is then an essential foundation [14]. Standardised pre-processing strengthens interoperability between devices and facilitates reproducibility of algorithm implementation. It lowers costs, speeds up development, and lays the groundwork for regulatory approval, which in turn supports clinical adoption and reimbursement.

In this work, a pre-processing framework for wearable accelerometer measurements is described and tested (Fig 1).

**Fig 1:**
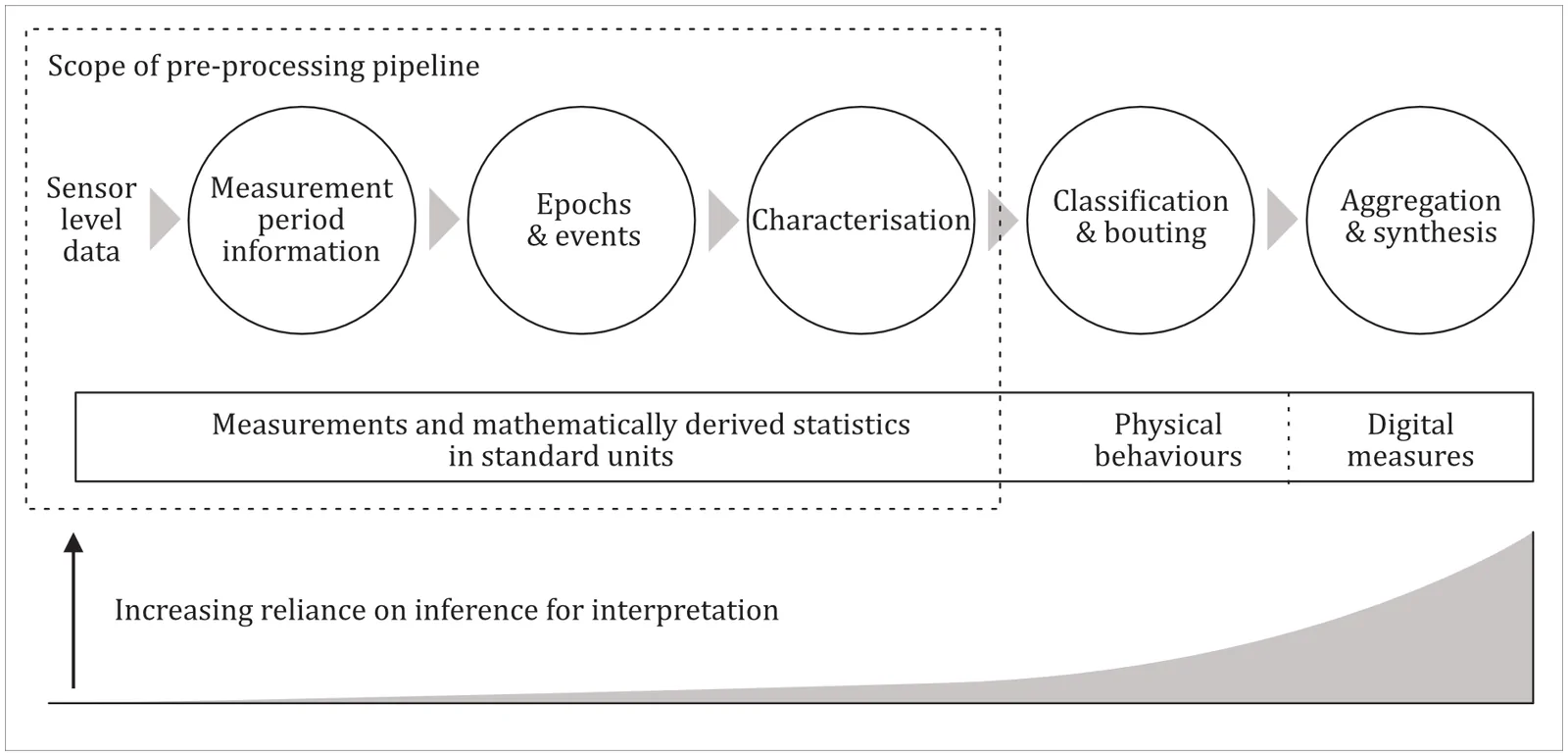
Generic sensor data processing pipeline. A representation of a generic sensor data processing pipeline showing the scope and aims of this work in the context of classified physical behaviours, digital measures and the level of inference required for interpretation.

The framework uses open-source software in an integrated test environment to extend verification from the original device measurements through to the digital measures output from the system. It is a fast, flexible layer for the implementation, assessment and analytical validation of digital measures.

The scope of this work covers the first three stages of Fig 1, transforming raw sensor data into contiguous periods of pre-processed data, ready for classification into physical behaviours (for example, sleep or walking). Our objective in this early processing is to keep assumptions to a minimum, and to document them carefully when they are required. We want to create a transparent, reproducible and data-driven process to give the strongest possible foundation for more complex later stages. The acceleration measurements directly represent real-world behaviours, often in standard units (SI), preserving reproducibility and interoperability when heuristic or machine learning approaches are applied for analysing digital measures in the context of health outcomes.

The overall aim of the study is to define an open, modular pipeline (*GENEAcore*) for the pre-processing of high-resolution accelerometry that can assure the quality and traceability of raw, sensor-level data as they are successively refined into digital measures. The process must be transparent and efficiently deliver essential summary information concerning data source integrity, interoperability and quality. This manuscript documents the verification of the engineering implementation and the analytical validation of outputs against reference datasets.

### Pre-processing pipeline

There are several examples of processing pipelines for accelerometry data openly available for use by researchers. Some rely on heuristic assumptions (GGIR [15]) or large machine learning datasets (OxWearables [16]) while others are focused on integrating data from multiple devices (NiMBaLWear [17], SKDH [18]). They are fully integrated packages, designed to calculate complex measures and summary reports using a range of technical approaches. Yet, they are all configured to output final digital measures rather than to assist the comparison and development of measures in a verified software environment which can satisfy the needs of clinical regulators [19]. They do not support variable-duration bout characterisation from raw data and can have opaque rules for managing imputation and interruptions. All have the common pre-processing elements of calibration, non-wear detection and basic measure characterisation which have been simplified into the *GENEAcore* package.

Returning to the first stage shown in Fig 1, the measurement period information contains all essential metadata including directions for correct file reading, unique data source identifiers to link records, calibration of the data to maximise sensitivity, determination of data completeness and the assessment of non-wear. The accurate determination of wear time is critical to understanding both participant adherence and data loss due to operational constraints [20]. A review of 14 non-wear detection algorithms has provided a consolidated approach with good accuracy and time resolution (S1 Table). This study demonstrates the traceability of file handling, the performance of the non-wear detection approach and formally validates a widely used threshold for device non-movement.

The second step in the pipeline is the decomposition of continuous time series data into a contiguous series of time windows. This splits the sensor-level data into manageable chunks for characterisation, analysis and visualisation. Fixed-duration epochs are traditionally used as they were simple to implement on early wearables and are convenient for researchers [21]. However, there is no intrinsic relationship between the events and the physical behaviours under investigation because the timing is arbitrary. The selection of the epoch duration is also difficult; too short and there is not enough data to characterise a behaviour effectively, too long and they contain multiple behaviours. Changes in estimates of time spent at various activity levels with different epoch lengths are well reported [22,23] acknowledging interactions with activity level cutpoints [24] and the subsequent impacts on public health guideline adherence [25] and associations with related outcomes [21,26]. Variable duration events [27] based on transitions [28] identified from the variability of the data itself gives an alternative approach. This method has the advantage of ensuring that different, consecutive behaviours are not summarised into a single time window – improving accuracy and reducing confusion in later classification stages.

The concept of a bout was originally used in public health guidelines based on a minimum duration of physical activity that was linked to health benefits [29]. Increasingly, bouts are used to analyse all types of physical behaviours (for example, movement patterns [30]) with variable minimum durations [31,32] and different bout criteria [33]. When working with epochs, bouts need to be reconstructed with, often, complicated rules for epoch inclusion and allowances for interruptions. Events provide a more natural transformation to behavioural bouts but may identify more interruptions than long epochs depending on the minimum event duration specified. In this study, optimal parameters for data-driven event detection are determined and the resulting physical behaviour event durations are investigated. The potential of event approaches to help resolve the challenges with epoch length selection and bout rules are discussed.

In the third step of Fig 1, the sensor-level measurements are processed to characterise the epoch or event. This involves the application of mathematical operations to each of the individual sensor measurements to describe the data in some way. Often, several features (ideally, independent) will be extracted for a multi-dimensional perspective. There are many standard, open algorithms that can be applied [34], and the pipeline flexibly supports the verified implementation of novel, raw data functions. This study will show the utility of the framework by comparing two commonly used techniques for estimating activity intensity in both the epoch and event paradigms.

The pre-processing pipeline is designed to establish consistent inputs for the remaining two steps beyond the scope of this work. Digital measures may be based directly on the data characteristics at this stage (for example, mean activity intensity) or rely on further manipulation and modelling to infer physical behaviours that would be recognisable to an observer (for example, sleep or walking). Ultimately, these measures are often aggregated into summaries of daily living activities over multiple days to represent the lifestyle of the participant (for example, total active duration).

Without pre-processing as a distinct and independent layer, the validation of a digital measure becomes inseparable from its base assumptions and calculation processes. A digital measure then cannot be compared independently of the package that created it. The risk is that one becomes locked into a plurification of end-to-end processing pipelines, such as those mentioned earlier, for every digital measure or have different interpretations of an individual’s physical behaviours depending on the pre-processing used.

## Methods

The study used triaxial acceleration and temperature data from the GENEActiv Original (ActivInsights Ltd, Kimbolton, United Kingdom) in the raw output ‘.bin’ format that contains all measurements and recording information. The GENEActiv (firmware versions 1.0, 1.30, 4.08a & 06.17) is a wrist-worn accelerometer device with +/-8*g* range and 3.9m*g* resolution with a + /-1 °C near-body temperature sensor. Acceleration is commonly expressed in milli-units of gravity, m*g*, where *g* is equal to 9.81 m/s^2^. The data files were processed in the open-source R package developed through this study, *GENEAcore 1.2.0* [35], hosted by CRAN (The R Foundation, Vienna, Austria). The work was completed by a cross-functional team of academic, industry and specialist software personnel.

Engineering verification of the *GENEAcore* software was completed through unit and integration testing with design controls (as described in ISO62304 [36]) and algorithm code inspection by relevant experts in the team. Development for the package was undertaken across Windows and macOS utilising R Studio and its associated R package development libraries *dev tools*, *usethis* and *testthat*. Source code control, package building and continuous integration testing were performed using GitHub and GitHub Actions. Unit testing utilised independently calculated values or reference results from previously published calculations and packages providing 90% code coverage by file. Builds were tested on Windows 11, macOS 26 and Ubuntu Linux 24.04 LTS. Package build and final release test approval is completed by automated build routines in clean, independent environments. The processed x, y and z acceleration signals and timestamps were compared with the corresponding outputs from the *GGIR 3.2–6* [37] and *GENEAread 2.0.10* [38] R packages (also available from CRAN) to confirm broad agreement between the packages.

Verification testing of the engineering implementation was performed using multi-day, wrist-worn datasets totaling 100 files from 68 participants and 69 unique devices (one participant wore two devices). The HeLP dataset was randomly selected from a school-based intervention study aiming to prevent obesity in children [39]. The SafeHeart dataset was randomly selected from an observational study in adults with implanted cardioverter-defibrillators [40]. The first author dataset was self-collected sequential wear periods that allowed within-device variability to be examined. Analytical validation testing of functional performance was completed with objective and observed criterion measure data for 36 participants from 65 files. Laboratory-generated datasets were created with 104 simulated posture changes over a 12-hour period with an even coverage of device orientations. The sleep dataset was collected with polysomnography (PSG) in a sleep clinic setting [41]. In the office-collected non-wear trial, participants were instructed to wear or place the device on their desks at specific times by an observer who recorded the times and non-wear durations. The datasets were selected from available resources to provide a sufficient volume of data for verification activities, also including a wide range of ages, a reasonable gender balance and different sampling frequencies to ensure generalisability (Table 1).

**Table 1:** Verification and validation datasets.

Dataset	Measurement period & data	Number of files & [part.]	Mean age & [range]	Male %
**Verification testing**				
Randomly selected participants from the HeLP study (non-dominant wrist)	9 days @ 85.7 Hz	33 [33]	9.75 yrs [9–10]	49%
Randomly selected participants from the SafeHeart study (wear side not specified)	17 days @ 50 Hz 30 days @ 20 Hz	34 [34]	64 yrs [21-84]	81%
Sequential recording from the lead author of this study (left, non-dominant wrist)	15 days @ 100 Hz	33 [1]	49.5 yrs [49–50]	100%
**Analytical validation**				
Laboratory-generated multi-orientation datasets (wear side not applicable)	12 hours @ 100 Hz 12 hours @ 10 Hz	2 [n/a]	n/a	n/a
Nights with simultaneous sleep PSG (both left and right wrist)	1 night @ 85.7 Hz	55 [28]	44.9 yrs [21–72]	61%
Office-collected non-wear (side of wear not specified)	8 hours @ 100 Hz	8 [8]	33.9 yrs [24–48]	25%

Descriptions of the data and participants (part.) used in the verification testing and analytical validation.

Ethical approval for the HeLP study was obtained from the Peninsula College of Medicine and Dentistry in March 2012 (reference number 12/03/140). The Institutional Review Board and Regional Ethics Committees of Copenhagen University Hospital Rigshospitalet and Amsterdam University Medical Centre approved the SafeHeart study protocol. Permission has been granted by the first author for the use of personal data for this study. The polysomnography studies were approved by the University College London ethics committee and the NRES Committee North East Sunderland ethics committees. ActivInsights have authorised the use of the office-collected non-wear data and confirmed it was collected with consent under appropriate safeguards.

All data processing and statistical tests were processed in R version 4.4.2 [42].

### Measurement period information

The measurement period information is initially extracted from a bin file by the *GENEAcore* package, which then implements an optimised file read procedure to extract 1-second downsampled time series data (a single sampled measurement in each second) for calibration, non-wear detection and transition detection. The measurement period information consists of the original meta-data from the recording, file management parameters and a record of all pre-processing steps (see S2 Table).

### Calibration

A rolling standard deviation is calculated from the 1-second downsampled accelerations over a defined window of 120 seconds [43], with a threshold of 13m*g* [44] applied to the 3 axes mean identifying measurements of device non-movement. These selected measurements are iteratively fitted to a sphere to estimate auto-calibration figures from real world data [45]. The values selected for all processing parameters are shown in S3 Table with references.

All multi-day files are auto-calibrated using this method with the number of iterations required and the improvements in calibration, as calculated by residual error, recorded. The laboratory-generated multi-orientation datasets, each with 53 periods of non-movement over 12 hours, are used to evaluate the correct convergence of the auto-calibration. Once the calibration was completed, the scale and offset elements are systematically perturbed and the residual error calculated (+/- 1% scale and +/- 50% offset).

### Non-wear detection

Many approaches to the detection of non-wear have been published since the advent of raw data acceleration measurement and a summary of algorithms is shown in S1 Table. The existing methods were assessed by the data features they used, the minimum detectable duration of non-wear, their time resolution, wear location and the criterion measures used for validation. These algorithms are normally for single devices although dual device systems are known [46]. The methods use features derived from acceleration across three axes in varying combinations with temperature and, occasionally, time of day.

While signal properties can change between device and sensor models, the standard deviation of acceleration appears to offer a consistent level of detection performance robust to poor calibration [47]. Low pass signal filtering, including epochs and running means, allow absolute values of acceleration to be used [48,49] and posture changes to be determined [50,51]. However, using filtered signals in subsequent analysis reduces dynamic range [52]. Some publications note that the axes can have different noise characteristics and temperature dependencies [53,54], suggesting they are best managed independently.

The temperature sensors within devices can be used with absolute thresholds [49,53,55,56], but have complex signal characteristics over time. While the thermal lag due to the mass of the wearable would suggest a first order response, the initial rate of change is suppressed by the internal hysteresis of the sensor and quantization of signal output [57]. Publications report seeing maximum changes in temperature on device removal of 2–3 °C after 3–5 minutes [43,49,58] and a decoupling from body temperature after 16 minutes [55]. These findings help to define an optimal time window for detecting device removal using temperature changes.

Earlier algorithms all use fixed length epochs with varying overlaps but, more recently, event-based approaches have been deployed with transition detection of the acceleration [49,59] and temperature signals [43,58]. Event-based approaches have the advantage of precise output time resolutions without needing to use very short epoch lengths which are prone to classification error [60].

Several algorithms use ‘boarding rules’ to update the assessment of non-wear events and neighbouring events once an initial classification has been made. These can be based on the duration of the non-wear events [50,61], the duration of the gaps between non-wear events [59] or the signal characteristics of the event [62]. In addition, these approaches can be combined with sleep determination [50,62], recognising this as the most likely behavioural confusion. Different rule sets are applied during night periods or when proximal events are determined to be sleep.

The increased performance levels of newer approaches (resolution, accuracy and minimum detection periods) have come at the expense of processing complexity. The *GENEAcore* non-wear algorithm aims to simplify and reduce processing times without compromising accuracy. Importantly for interoperability and modularity, the detected non-wear periods are stored in the metadata separately from the time series measurements. It uses approaches, features and parameter values that have been successfully and consistently used in the prior art described above (S1 Table and S3 Table). It is an event-based approach with candidate events identified when the previously derived, 2-minute rolling average of 1-second downsampled acceleration standard deviation drops below 13m*g* [44]. Boarding rules are used on candidate events to combine events that are separated by less than 5 minutes with each added event counted a posture change. These events are classified as non-wear when any of these rules are satisfied:

- Temperature drop due to device removal – more than 0.7 °C per minute drop in the first 4 minutes of device non-movement with less than two posture changes; or
- Sustained device non-movement without posture change – any single event that is greater than 2 hours in duration; or
- Long-running device non-movement – continuous device non-movement periods greater than 12 hours in duration irrespective of the number of posture changes.

The resulting outputs have a minimum detectable non-wear event duration of 5 minutes (timespan of temperature drop rule plus rolling average lag) with a time resolution of 1 second. These are stored in the measurement period information.

Here, we test the non-wear algorithm with wrist-worn data against both the observer diaries of controlled non-wear and sleep clinic participation. The office non-wear and sleep polysomnography criterion data are processed to create estimates for non-wear for each second. The estimates are then matched with observer records to ascertain true positive, true negative, false positive and false negative rates which are combined into balanced accuracy (sensitivity/2 + specificity/2). A sensitivity analysis investigated the impact of changes in the acceleration standard deviation threshold on the classification accuracy.

### Epochs and events

Epochs and events are specified by their integer second start time and duration. Epochs of any integer duration can be selected, and the sequence starts from the beginning of the first minute of the day. The transition detection for event start times utilises the 1-second downsampled time series extracted with the measurement period information.

### Transition detection

*GENEAcore* uses Pruned Exact Linear Time (PELT) changepoint detection [63] from the *Changepoint 2.2.2* [64] R package to identify transitions in the mean and variance of downsampled acceleration. Each axis is assessed separately with the resulting timestamps combined while amalgamating transitions within 5 seconds of each other (minimum event duration).

The PELT method uses a penalty value to control sensitivity to changes in the data and, therefore, the average number of transitions detected. Accelerometer studies in animals have previously used sample entropy to select penalty values that maximise the information density of the event summary [65]. The total root mean square error (RMSE) between the event and 1-second epoch acceleration intensity is calculated on each axis for different penalty values on a day randomly selected from each of the 100 verification files. Inflection points in the relationship between the average number of events per day and RMSE represent the point where a stable representation of the original signal has been reached. The purpose is to select penalty values appropriate for accelerometer data generally, rather than the tuning for the detection of specific behaviours, and this was done through visual inspection.

Once suitable penalty values are selected for each axis, the two multi-orientation laboratory datasets are used to test the method. The timings of true transitions are compared to those determined by the PELT algorithm. The impact of varying the minimum event duration on the average number of events per day for each verification dataset is then calculated. Additionally, the distribution of event durations is investigated and the ratio of event to 1-second epoch data volumes (compression ratio) is determined.

### Characterisation

At this point in the processing, the measurement period information contains sensor calibration parameters, non-wear periods and event transitions. The raw, sensor-level measurements can now be processed as events or epochs with the selected algorithms to characterise the data. Any further processing to classify the events or epochs (for example, as sleep or high intensity activity) is a further step beyond the scope of pre-processing (Fig 1).

The architecture of the *GENEAcore* package allows different methods to be executed within a consistent processing environment. Here, two activity intensity measures that subtract gravity from the acceleration signal and two different approaches to aggregation are compared.

The earliest raw acceleration summary algorithm [66] took the sum of the gravity-subtracted vector magnitudes in an epoch (SVM_gs_) using the absolute value of any negative numbers. The resulting measure can be difficult to interpret as it is linearly dependent on both the sample frequency and epoch length; the mean of the absolute gravity subtracted acceleration (AGSA) [67,68] addresses this drawback. Euclidean norm minus one (ENMO) was later proposed [61] and differs only by setting negative numbers from the subtraction of gravity to zero.

AGSA and ENMO are calculated for all time points in the verification dataset using 1-second epochs as this is recognised to give the most accurate assessment of instantaneous intensity [69,70]. The overlapping coefficient of the distributions above and below the 13m*g* standard deviation movement threshold were calculated for each measure to understand their ability to discriminate effectively in low movement conditions. AGSA and ENMO are then aggregated by day to investigate the concordance [71,72] of assessed active duration using a previously defined AGSA threshold of 62.5m*g* [66,73] and the modelled ENMO equivalent.

Despite the absence of an intensity criterion measure, it is still possible to illustrate how an independent pre-processing stage supports future research. Event and 1-second epoch outputs are also calculated for each day in the verification dataset with active periods identified using the AGSA 62.5m*g* threshold. Both are then aggregated by day to investigate the differences between variable duration event and fixed duration 1-second epochs aggregation on estimates of daily active duration.

## Results

The *GENEAcore* pre-processing pipeline was configured to output three data objects: the measurement period information, 1-second epochs and variable duration events. Once completed, the measurement period information contained calibration values and non-wear periods separated from both the original data source and output time-series data (epochs and events). These objects were traceably linked by a unique source identifier derived from source file itself, rather than any manually controlled file name.

### Calibration

During auto-calibration of the verification datasets, the mean number of iterations required to meet the incremental fit improvement threshold (see S3 Table) was 51.3 (31.6 SD). The mean improvement from factory calibration was 9.5m*g* resulting in a mean residual sphere fit error of 6.7m*g* (0.48m*g* SD).

All calibration perturbations of the laboratory-generated multi-orientation datasets resulted in an increased residual sphere fit error, confirming convergence. The mean increase for a 1% change in scale was 1.1m*g*, and 1.2m*g* for a 50% change in offset.

The repeatability of the auto-calibration was evaluated on the two devices worn multiple times by the lead author. The mean standard deviation of calibration gain and offset between devices was 19.5m*g* and 31.5m*g*, while the within device mean standard deviation was 1.0m*g* and 2.1m*g*, respectively.

### Non-wear detection

The sleep polysomnography (PSG) and office non-wear criterion data were processed to create estimates of non-wear for each second. The office non-wear participants were each observed to have an average of 7.5 non-wear events with a mean duration of 1470 seconds (min. 178, max. 3357), interspersed by wear periods with 1046 second mean duration (min. 219, max. 3549). All assessments of non-wear during polysomnography measurements were assumed to be false positives. Table 2 shows the resulting sensitivity, specificity and balanced accuracy alongside the base observation rates as described in the methods.

**Table 2:** Non-wear detection performance.

	True positives	True negatives	False positives	False negatives	Sensitivity	Specificity	Balanced accuracy
Sleep PSG	0	1.8x10^6^	906	0	—	99.95%	—
Office non-wear	66564	118240	2326	11918	84.8%	98.1%	91.4%
Combined	66564	2.0x10^6^	3232	11918	84.8%	99.8%	92.3%

Results of non-wear detection algorithm testing in different wear scenarios.

The acceleration standard deviation threshold of value was varied between 6m*g* and 20m*g* to test whether the widely used 13m*g* threshold [44] for identifying device non-movement was optimal for this sensor and non-wear algorithm in these wear scenarios. The changes in detection performance are shown in Fig 2, where a marginal increase in balanced accuracy from 92.3% to 92.6% can be seen at an acceleration standard deviation threshold of 12m*g*.

**Fig 2:**
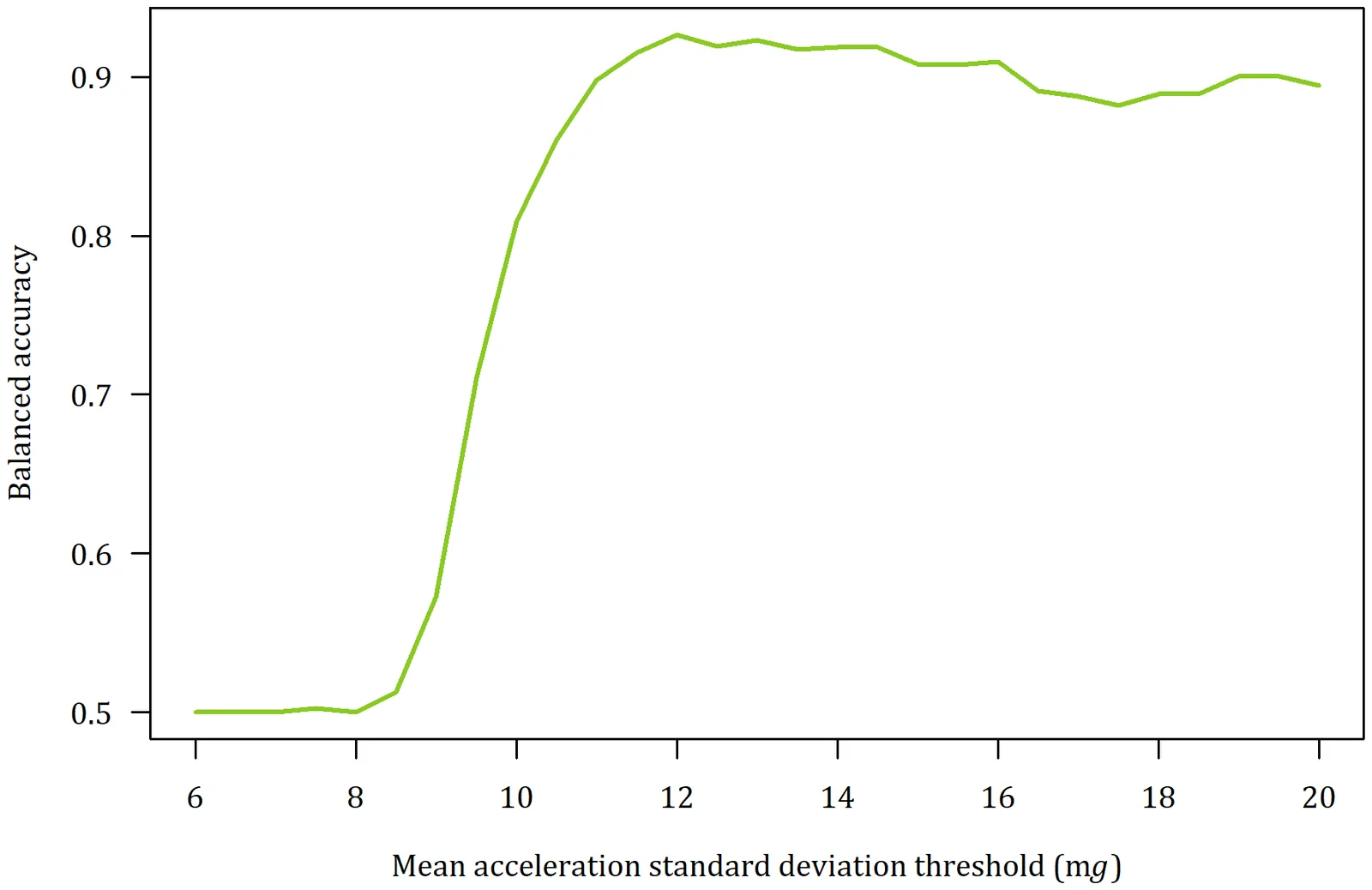
Non-wear detection sensitivity analysis. Changes in non-wear detection performance as measured with balanced accuracy for different values of mean acceleration standard deviation threshold.

### Transition detection

Optimal penalty values for PELT changepoint detection were found to be 18, 25 and 16 for the x, y and z axes respectively. When validated against the two multi-orientation datasets, only 1 of 104 transitions was not correctly identified and mean minimum distance to transitions was 1.65 seconds.

For each axis individually, the target number of events per day, based on the RMSE inflection points, was approximately 500. When the detected transitions for each axis in the verification dataset were combined with a minimum event duration of 5 seconds, the mean number of events per complete day (number of days = 1,296) was 1,116 (221 SD). S1 Fig shows plots of RMSE, events per day and penalty value with inflection points for each axis and verification dataset sub-group. The minimum event duration of 5 seconds was selected as the shortest possible event duration that could occur between two transitions each lasting 3 seconds [28].

The minimum permitted event duration when combining transitions was varied from 3 to 60 seconds with plots of resulting transitions per day for each verification dataset sub-group shown in Fig 3. The number of events per day reduces as more shorter events are combined. The rate of change of decline is similar for all verification datasets, starting slowly and then increasing for values above 10 seconds.

**Fig 3:**
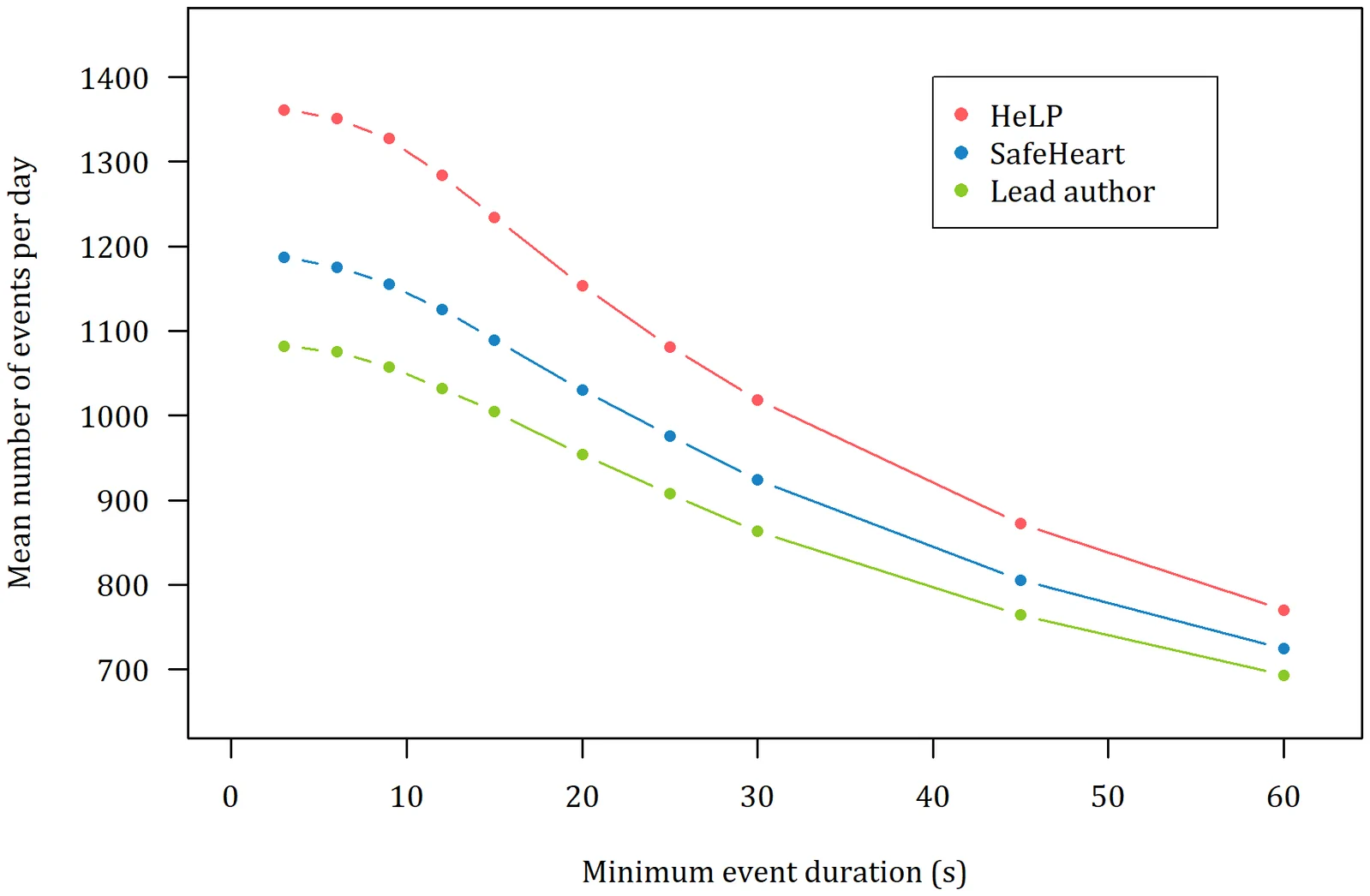
Impact of minimum event duration on events per day. The variation of the number of daily events with minimum event duration in the three verification population groups.

The distribution of event durations approximated closely to the log normal (μ = 3.9, σ = 1.1) giving an expected event duration, E(X), of 68.6 seconds.

Data summaries based on variable length events provided a compression ratio of 87.9 compared to a 1-second fixed epoch output, while preserving a 1 second resolution for changes in the underlying data.

### Characterisation

The relationship between AGSA and ENMO is shown in Fig 4 for every second in the verification dataset (130,484,716 data points). The red group has all time classed as moving (mean standard deviation of acceleration above 13m*g*) and the blue group contains the remaining time classed as non-movement. Above 1*g* of mean acceleration, AGSA and ENMO give near identical results. In the lower acceleration ranges of the moving group, ENMO consistently under-reports compared to AGSA. In the non-movement group, the asymmetric treatment of negative values in the ENMO algorithm is apparent, with values of mean acceleration often reported below 1m*g* despite the 6-7m*g* noise floor of the sensor. The overlapping coefficient of AGSA distributions of moving and non-movement groups was 0.053 while the ENMO overlap was 0.098.

**Fig 4:**
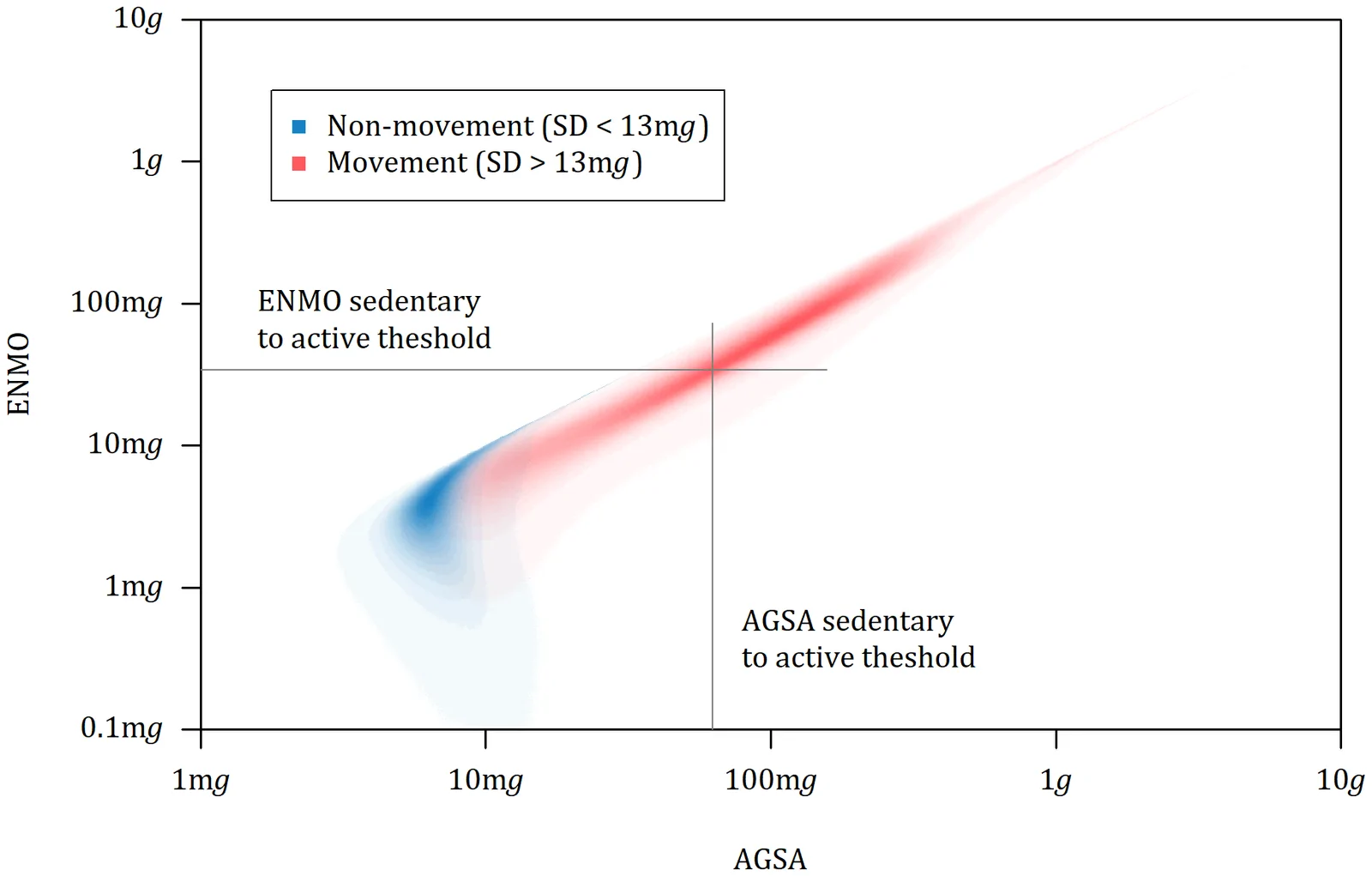
Comparison of activity intensity calculated by AGSA and ENMO. Density plot of 1s epochs summarised by AGSA and ENMO for the verification dataset split into groups of non-movement (blue) and movement (red).

The typical sedentary to active threshold for AGSA of 62.5m*g* [66,73] is shown vertically in Fig 4. A linear regression of the movement group (r^2^ = 0.957, slope = 0.872, intercept = −20.5 m*g*) establishes an equivalent threshold for ENMO of 34.1m*g*, shown horizontally.

When these sedentary to active thresholds are applied daily over 1,296 complete days, the concordance of the resulting daily active duration was found to be greater than 0.99. Correlation and Bland-Altman plots are shown in S2 Fig. ENMO has bias of +1.25 minutes per day compared to AGSA.

The *GENEAcore* pre-processing pipeline allows us to contrast event and epoch segmentation. The correlation of active duration per day determined with variable duration events and fixed duration 1-second epochs is shown in S3 Fig with an accompanying Bland-Altman regression plot. The concordance was 0.831 (95% CI, 0.821–0.842), however the Pearson correlation coefficient was 0.985 (95% CI, 0.983–0.986). The regression model of the differences shows the event-derived active durations per day to be consistently 31.5% (95% CI, 30.8–32.2) higher than those from 1-second epochs.

## Discussion

The aim of this study was to define and test a simplified, modular pipeline for the pre-processing of high-resolution accelerometry – assuring the quality of raw, sensor-level data as they are successively refined into digital measures.

The handling of data objects during the study demonstrated full traceability from input data sources to the output digital measures with a record of all processing steps in the measurement period information. The modularity of implementation and reporting provides transparency, while also supporting easy application of new or improved techniques. Integrated testing and change control will assure regulators of the suitability for health applications.

Although the pipeline software has been written in R to read data files generated by the GENEActiv accelerometer device, the methods and results have wide applicability. The open-source code will allow engineers to reproduce functions for other accelerometer devices in other programming languages. Algorithms are not wear location specific and have been described with all processing parameters transparently available. Any learnings about the differences between intensity measures and variable-length events versus fixed-length epochs are independent of the measurement device.

Results recorded for the calibration stage confirm the success of earlier studies [45], showing residual error comparable to the noise floor of the sensor itself. Furthermore, the perturbation tests demonstrate correct convergence of the optimised method and repeatability tests confirm that the calibration of these sensors is static over time. The detection of transitions using changepoint analysis proved reliable with 99% of transitions detected, on average, within 2 seconds of their occurrence.

This is the first study to empirically test the 13m*g* acceleration standard deviation threshold commonly used in the literature since 2014 [44] with a sensitivity analysis. This was achieved by calculating the balanced accuracy of non-wear detection through a range of threshold values. The results indicate a slight reduction of the threshold could be beneficial for this specific device, however preserving current practice affords some leeway for noisier sensors, with a minimal reduction in efficacy.

The non-wear algorithm components, all based on existing know-how, are independent of body location and show no variation between adults and children or male/ female participants [55]. We consider that the approaches and results have high generalisability to wear position and population, suited to real-time adherence monitoring due to the processing simplicity. The criterion datasets used to test the non-wear algorithm were selected to challenge in situations where performance can be marginal: sleep measured by PSG and short periods in a lived environment controlled by an observer. Of the previous non-wear validation studies, two used observers [50,62], one used a separate device [59] all others relied on participant diaries or expert rating of data. The high overall specificity is excellent for sleep measurement where false positives for non-wear during the night are problematic for clinicians. The sensitivity to short off-wrist periods is reasonable; it will be higher and comparable to the other published methods for longer periods of non-wear where the participant is not in close proximity to the device. The detection of non-wear during the transport of devices to participants will remain difficult, however this is normally accompanied by long, still periods where accuracy is high.

The detailed comparison of two commonly used measures of activity intensity (AGSA and ENMO) reveal that attention must be given through every stage of digital measure selection and embodiment for any health application. A small change in the algorithm has major consequences. The sedentary AGSA threshold of 62.5m*g* [66,73] was translated to an equivalent ENMO threshold of 34.1m*g*; a value which is exactly in line with other studies across a range of populations [74,75]. Therefore, for higher movement levels above the sedentary thresholds, AGSA and ENMO are substitutable for measuring daily active times using existing thresholds or the linear regression described. More care is required at lower movement levels, when analysing individual events and when using activity intensity as a continuous measure. Below the sedentary threshold, ENMO quickly becomes non-linear with reduced movement, outputting results inconsistent with the measurement noise of the sensor. There is more headroom for AGSA below the sedentary threshold, as indicated by the lower overlapping coefficient, until sensitivity is lost in a more linear fashion.

A particular contribution of this work is the comparison of epoch and event pre-processing made possible by the *GENEAcore* pipeline. Variable duration events created higher assessments of daily activity duration compared to those calculated from 1-second epochs. The disparity in outputs was an extra 19 minutes for every additional hour of activity. The magnitude of this disparity will vary with the epoch length selected for comparison. The threshold used in this analysis to identify active behaviours encompasses light, moderate and vigorous intensity physical activity. Longer epochs are consistently reported to reduce estimates of both vigorous and sedentary time [23–26,76], pushing all estimates towards the mid-intensity bands. So, the disparities in reported active time compared to events will decrease with longer epochs. However, in the epoch paradigm, these longer epochs will under-report vigorous active times, whereas variable duration events will still capture short bursts of higher intensity physical activity. This ability of event-based processing to adapt to the latent structures of the data provides a solution to suggestions that different epoch lengths are more effective at detecting behaviours of different intensities [21,22].

Data-driven estimates of the typical durations of human movement behaviours are not widely available. The log normal distribution found here, with an expected event duration of just over a minute, is similar to that of other land-based mammals [65]. This result will support the modelling of event durations in further studies. A minimum event duration of 5 seconds preserved the complexity of daily data, allows short bouts of activity to be recorded [76,77] and gives a finite time for the transitions themselves [28]. These findings confirm the existing conventions of epoch length selection of between 1 and 60 seconds. The compromises associated with epoch length choices outlined above are resolved by variable duration encoding. The event-based paradigm delivers the timestamp accuracy of short epochs and efficient data compression of longer epochs with high classification confidence. In addition, the relative timings of the transitions themselves expose underlying patterns of physical behaviours.

The uncertainties associated with the selection of appropriate epoch length are compounded once epochs are grouped to represent bouts of physical behaviour. The *GENEAcore* pre-processing pipeline specifies event transitions prior to characterisation and classification – allowing an explicit translation from data-driven events to bouts without further processing. In contrast, existing epoch-only pipelines must apply additional rules with parameters to identify bout transitions, epoch aggregation and permissible interruptions. These rules require substantial verification and analytical validation for them to be used repeatably and with confidence.

At first glance, event approaches appear more complicated than the use of traditional epochs. The variable durations pose methodological problems in parameter selection, visualisation, aggregation and bridging of reporting boundaries (for example, across days). However, these obstacles are inherent to bout representations and data-driven event transition detection simplifies the later stages of process validation. Lastly, events defined in the early steps of pre-processing supply event duration as a valuable, additional feature for later classification stages.

This study has limitations in the small sizes of datasets used for the analytical validation steps, absence of processing time comparisons between existing packages and the lack of a criterion measure for activity intensity. The selection of penalty values for the changepoint analysis was completed by inspection rather than through an objective process using only wrist-worn data. Additional work could explore the sensitivity of penalty values, other techniques for determining appropriate values and other impact of wear locations. It was not possible, within this study, to test the non-wear algorithm in a wider range of populations that included children where previous algorithms have had sub-optimal performance [50].

Future work can use other larger, previously collected data to further investigate comparative accuracies of AGSA vs. ENMO activity intensities and epoch vs. event pre-processing. Ideally, analysis work would use these as continuous measures of activity intensity rather than cutpoint-derived energy expenditure classes to avoid threshold effects. A better understanding of the impact of the rules for bout creation from epochs on the reporting of physical activity acquisition (durations, volumes and patterns) at different epoch lengths is needed. Data-driven events would provide a stable benchmark for this analysis. Study strengths include the use of a cross-functional team to create a maintainable, engineered tool with backward compatibility for existing data. Deliberately constraining scope to the pre-processing pipeline alone enhanced the depth and focus of results. The thorough documentation of assumptions will support the consistent implementation of existing measures identified as important to public health [78,79] as well as clinical research.

## Conclusions

The focus on verification and analytical validation of accelerometer pre-processing has exposed detailed engineering considerations that must underpin reliable digital measures. The rush towards results with health associations often leaves little room for these important topics.

The meticulous evaluation of the simplest steps of pre-processing has confirmed the attention to detail that is required to create robust and reproducible results in later clinical validation. The integrity of data pathways is preserved through standardised approaches and rigorous implementation.

We have shown this optimised framework to be a capable building block of future research, clinical care and public digital health systems. It can deliver the basic digital measures needed to understand physical behaviours over multiple days with minimum assumptions.

## Supporting information

S1 Table Non-wear detection methods. Descriptions of different methods for the detection of non-wear including: the general method, features used for detection, the minimum detectable non-wear duration, the time resolution of non-wear periods, the criterion used in their validation and the wear location.
(PDF)

S2 Table Measurement period information. The data structure of the original meta-data from the recording, file management parameters and record of all pre-processing steps.
(PDF)

S3 Table Processing parameters. The definitions, values and the supporting references for the parameters used in the pre-processing operations.
(PDF)

S1 Fig Changepoint penalty value assessments. The variation of the number of daily events and the RMSE of intensity with changepoint penalty value for each axis of acceleration in the three verification population groups.
(PDF)

S2 Fig Comparing daily active duration using AGSA and ENMO. (a) Correlation plot of active hours per day as assessed by AGSA and ENMO, (b) Bland-Altman plot of the means and differences of AGSA and ENMO active hours per day with mean bias and 95% SD confidence intervals.
(PDF)

S3 Fig Comparing daily active duration using event and epoch aggregates of AGSA. (a) Correlation plot of active hours per day as assessed by AGSA 1s epoch and variable duration events, (b) Bland-Altman regression plot modelling the differences of AGSA 1s epoch and variable duration event active hours by event-assessed duration per day with mean bias and 95% SEE confidence intervals.
(PDF)

## References

[1] Liu F, Wanigatunga AA, Schrack JA. Assessment of Physical Activity in Adults Using Wrist Accelerometers. Epidemiol Rev. 2022;43(1):65–93. doi: 10.1093/epirev/mxab004 34215874 PMC8900289

[2] US. Food and Drug Administration. Digital Health Technologies for Remote Data Acquisition in Clinical Investigations. U.S. Food and Drug Administration; 2023.

[3] Canali S, Schiaffonati V, Aliverti A. Challenges and recommendations for wearable devices in digital health: Data quality, interoperability, health equity, fairness. PLOS Digit Health. 2022;1(10):e0000104. doi: 10.1371/journal.pdig.0000104 36812619 PMC9931360

[4] Goldsack JC, Coravos A, Bakker JP, Bent B, Dowling AV, Fitzer-Attas C, et al. Verification, analytical validation, and clinical validation (V3): the foundation of determining fit-for-purpose for Biometric Monitoring Technologies (BioMeTs). NPJ Digit Med. 2020;3:55. doi: 10.1038/s41746-020-0260-4 32337371 PMC7156507

[5] Guan G, Mofaz M, Qian G, Patalon T, Shmueli E, Yamin D, et al. Higher sensitivity monitoring of reactions to COVID-19 vaccination using smartwatches. NPJ Digit Med. 2022;5(1):140. doi: 10.1038/s41746-022-00683-w 36085312 PMC9461410

[6] Aunger J, Wagnild J. Objective and subjective measurement of sedentary behavior in human adults: A toolkit. American J Hum Biol. 2020;34(1). doi: 10.1002/ajhb.23546 PMC9286366 33277954

[7] Prince SA, Cardilli L, Reed JL, Saunders TJ, Kite C, Douillette K, et al. A comparison of self-reported and device measured sedentary behaviour in adults: a systematic review and meta-analysis. Int J Behav Nutr Phys Act. 2020;17(1):31. doi: 10.1186/s12966-020-00938-3 32131845 PMC7055033

[8] Tully MA, Panter J, Ogilvie D. Individual characteristics associated with mismatches between self-reported and accelerometer-measured physical activity. PLoS One. 2014;9(6):e99636. doi: 10.1371/journal.pone.0099636 24919185 PMC4053373

[9] Guo W, Key TJ, Reeves GK. Accelerometer compared with questionnaire measures of physical activity in relation to body size and composition: a large cross-sectional analysis of UK Biobank. BMJ Open. 2019;9(1):e024206. doi: 10.1136/bmjopen-2018-024206 30700478 PMC6352868

[10] International Organization for Standardization. IEC 62366-1:2015. Application of usability engineering to medical devices. Geneva: ISO. 2015.

[11] European Commission. Clinical Evaluation: A Guide for Manufacturers and Notified Bodies under Directives 93/42/EEC and 90/385/EEC. MEDDEV 2.7/1 revision 4. Brussels: EC. 2016.

[12] International Organization for Standardization. ISO 13485:2016. Medical devices — Quality management systems — Requirements for regulatory purposes. Geneva: ISO. 2016.

[13] International Organization for Standardization. Medical devices — Application of risk management to medical devices. Geneva: ISO. 2019.

[14] Dibben GO, Santillan C, Brage S, Buman M, Duncan E, Granat MH, et al. Reporting of Accelerometry in Health Research: A Scoping Review of Current Guidance. Scand J Med Sci Sports. 2025;35(10):e70143. doi: 10.1111/sms.70143 41091538 PMC12525895

[15] Migueles JH, Rowlands AV, Huber F, Sabia S, van Hees VT. GGIR: A research community–driven open source R package for generating physical activity and sleep outcomes from multi-day raw accelerometer data. Journal for the Measurement of Physical Behaviour. 2019;2(3):188–96.

[16] Doherty A, Chan S, Yuan H, Walmsley R. Accelerometer: A Python Toolkit for Extracting Physical Activity and Behavior Metrics from Wearable Sensor Data. Zenodo. 2020. doi: 14515076

[17] Beyer KB, Weber KS, Cornish BF, Vert A, Thai V, Godkin FE, et al. NiMBaLWear analytics pipeline for wearable sensors: a modular, open-source platform for evaluating multiple domains of health and behaviour. BMC Digit Health. 2024;2(1):8. doi: 10.1186/s44247-024-00062-3 42529548 PMC13417704

[18] Lin W, Karahanoglu FI, Demanuele C, Khan S, Cai X, Santamaria M, et al. SciKit digital health package for accelerometry-measured physical activity: comparisons to existing solutions and investigations of age effects in healthy adults. Front Digit Health. 2023;5:1321086. doi: 10.3389/fdgth.2023.1321086 38090655 PMC10715430

[19] Murray ML, Love SB, Carpenter JR, Hartley S, Landray MJ, Mafham M, et al. Data provenance and integrity of health-care systems data for clinical trials. Lancet Digit Health. 2022;4(8):e567–8. doi: 10.1016/S2589-7500(22)00122-4 35868811 PMC9296098

[20] Frodi DM, Kolk MZH, Langford J, Knops R, Tan HL, Andersen TO, et al. Long-term adherence to a wearable for continuous behavioural activity measuring in the SafeHeart implantable cardioverter defibrillator population. Eur Heart J Digit Health. 2024;5(5):622–32. doi: 10.1093/ehjdh/ztae055 39318686 PMC11417489

[21] Gabriel KP, McClain JJ, Schmid KK, Storti KL, High RR, Underwood DA, et al. Issues in accelerometer methodology: the role of epoch length on estimates of physical activity and relationships with health outcomes in overweight, post-menopausal women. Int J Behav Nutr Phys Act. 2010;7:53. doi: 10.1186/1479-5868-7-53 20550691 PMC2900223

[22] Altenburg TM, Wang X, van Ekris E, Andersen LB, Møller NC, Wedderkopp N, et al. The consequences of using different epoch lengths on the classification of accelerometer based sedentary behaviour and physical activity. PLoS One. 2021;16(7):e0254721. doi: 10.1371/journal.pone.0254721 34265011 PMC8282067

[23] Lee J-H, Kwon S, Lee S, Jang S-Y, Jo W, Jin J, et al. The influence of accelerometer epoch length on physical activity output in adolescent athletes. J Exerc Rehabil. 2023;19(6):370–4. doi: 10.12965/jer.2346576.288 38188137 PMC10766452

[24] Burchartz A, Kolb S, Klos L, Schmidt SCE, von Haaren-Mack B, Niessner C, et al. How specific combinations of epoch length, non-wear time and cut-points influence physical activity. Ger J Exerc Sport Res. 2023;54(2):169–78. doi: 10.1007/s12662-023-00892-9

[25] Haddadj R, Verdot C, Salanave B, Deschamps V, Vanhelst J. Impact of varying accelerometer epoch length on physical activity patterns in adults: Considerations for public health. PLoS One. 2024;19(12):e0316176. doi: 10.1371/journal.pone.0316176 39739651 PMC11687789

[26] Aadland E, Nilsen AKO. Accelerometer epoch length influence associations for physical activity intensities with body mass index and locomotor skills in young children. J Sports Sci. 2022;40(14):1568–77. doi: 10.1080/02640414.2022.2092979 35758298

[27] Granat MH. Event-based analysis of free-living behaviour. Physiol Meas. 2012;33(11):1785–800. doi: 10.1088/0967-3334/33/11/1785 23110873

[28] Twaites J, Everson R, Langford J, Hillsdon M. Transition Detection for Automatic Segmentation of Wrist-Worn Acceleration Data: A Comparison of New and Existing Methods. Journal for the Measurement of Physical Behaviour. 2020;3(1):19–28. doi: 10.1123/jmpb.2019-0006

[29] Pate RR. Physical activity and public health. A recommendation from the Centers for Disease Control and Prevention and the American College of Sports Medicine. JAMA: The Journal of the American Medical Association. 1995;273(5):402–7. 10.1001/jama.273.5.402 7823386

[30] Kobeissi E, Dempsey P, Alshallal A, Rennie KL, Brage S, Wijndaele K, et al. A Systematic Review and Taxonomy of Metrics and Methods Used to Describe Temporal Movement Behavior Patterns Measured Using Wearable Devices. Journal for the Measurement of Physical Behaviour. 2026;9(1). doi: 10.1123/jmpb.2024-0035

[31] Ahmadi MN, Hamer M, Gill JMR, Murphy M, Sanders JP, Doherty A, et al. Brief bouts of device-measured intermittent lifestyle physical activity and its association with major adverse cardiovascular events and mortality in people who do not exercise: a prospective cohort study. Lancet Public Health. 2023;8(10):e800–10. doi: 10.1016/S2468-2667(23)00183-4 37777289

[32] Ma T, Sirard J, Yang L, Li Y, Tsang S, Fu A. Revisiting the concept of bout: associations of moderate-to-vigorous physical activity sessions and non-sessions with mortality. Int J Behav Nutr Phys Act. 2024;21(1):81. doi: 10.1186/s12966-024-01631-5 39075398 PMC11287937

[33] Eke H, Bonn SE, Trolle Lagerros Y. Wrist-worn accelerometers: Influence of decisions during data collection and processing: A cross-sectional study. Health Sci Rep. 2024;7(1):e1810. doi: 10.1002/hsr2.1810 38213780 PMC10782047

[34] Liang Y-T, Wang C, Hsiao CK. Data Analytics in Physical Activity Studies With Accelerometers: Scoping Review. J Med Internet Res. 2024;26:e59497. doi: 10.2196/59497 39259962 PMC11425027

[35] Chua JY, Langford J, Long IP. Re-processing of “GENEActiv” Data. R-project.org. 2026. https://www.r-project.org

[36] International Organization for Standardization. IEC 62304:2006. Medical device software — Software life cycle processes. Geneva: ISO. 2006.

[37] van Hees VT, Migueles JH, Afolabi ST. Raw accelerometer data analysis. R package GGIR version 3.2-6. https://R-project.org 2014.

[38] Chua JY, Langford J, Sweetland C, Fang Z. Package for Reading Binary Files. R-project.org. 2024.

[39] Wyatt KM, Lloyd JJ, Abraham C, Creanor S, Dean S, Densham E, et al. The Healthy Lifestyles Programme (HeLP), a novel school-based intervention to prevent obesity in school children: study protocol for a randomised controlled trial. Trials. 2013;14:95. doi: 10.1186/1745-6215-14-95 23556434 PMC3652736

[40] Frodi DM, Kolk MZH, Langford J, Andersen TO, Knops RE, Tan HL, et al. Rationale and design of the SafeHeart study: Development and testing of a mHealth tool for the prediction of arrhythmic events and implantable cardioverter-defibrillator therapy. Cardiovasc Digit Health J. 2021;2(6 Suppl):S11–20. doi: 10.1016/j.cvdhj.2021.10.002 35265921 PMC8890037

[41] van Hees VT, Sabia S, Anderson KN, Denton SJ, Oliver J, Catt M. A Novel, Open Access Method to Assess Sleep Duration Using a Wrist-Worn Accelerometer. PLOS ONE. 2015;10(11):e0142533. doi: 10.1371/journal.pone.0142533 PMC4646630 26569414

[42] R Core Team. R: The R Project for Statistical Computing. https://www.r-project.org 2025.

[43] Barakat A, Manga A, Sheikh A, McWilliams R, Rowlands AV, Singh H. Feasibility of Using a GENEActiv Accelerometer with Triaxial Acceleration and Temperature Sensors to Monitor Adherence to Shoulder Sling Wear Following Surgery. Sensors (Basel). 2024;24(3):880. doi: 10.3390/s24030880 38339597 PMC10856901

[44] da Silva IC, van Hees VT, Ramires VV, Knuth AG, Bielemann RM, Ekelund U, et al. Physical activity levels in three Brazilian birth cohorts as assessed with raw triaxial wrist accelerometry. Int J Epidemiol. 2014;43(6):1959–68. doi: 10.1093/ije/dyu203 25361583 PMC4276065

[45] van Hees VT, Fang Z, Langford J, Assah F, Mohammad A, da Silva ICM, et al. Autocalibration of accelerometer data for free-living physical activity assessment using local gravity and temperature: an evaluation on four continents. J Appl Physiol (1985). 2014;117(7):738–44. doi: 10.1152/japplphysiol.00421.2014 25103964 PMC4187052

[46] Syed S, Morseth B, Hopstock LA, Horsch A. Evaluating the performance of raw and epoch non-wear algorithms using multiple accelerometers and electrocardiogram recordings. Sci Rep. 2020;10(1):5866. doi: 10.1038/s41598-020-62821-2 32246080 PMC7125135

[47] van Hees VT, Renström F, Wright A, Gradmark A, Catt M, Chen KY, et al. Estimation of daily energy expenditure in pregnant and non-pregnant women using a wrist-worn tri-axial accelerometer. PLoS One. 2011;6(7):e22922. doi: 10.1371/journal.pone.0022922 21829556 PMC3146494

[48] Rahimi-Eichi H, Coombs Iii G, Vidal Bustamante CM, Onnela J-P, Baker JT, Buckner RL. Open-source Longitudinal Sleep Analysis From Accelerometer Data (DPSleep): Algorithm Development and Validation. JMIR Mhealth Uhealth. 2021;9(10):e29849. doi: 10.2196/29849 34612831 PMC8529474

[49] Vert A, Weber KS, Thai V, Turner E, Beyer KB, Cornish BF, et al. Detecting accelerometer non-wear periods using change in acceleration combined with rate-of-change in temperature. BMC Med Res Methodol. 2022;22(1):147. doi: 10.1186/s12874-022-01633-6 35596151 PMC9123693

[50] Ahmadi MN, Nathan N, Sutherland R, Wolfenden L, Trost SG. Non-wear or sleep? Evaluation of five non-wear detection algorithms for raw accelerometer data. J Sports Sci. 2020;38(4):399–404. doi: 10.1080/02640414.2019.1703301 31826746

[51] Sundararajan K, Georgievska S, Te Lindert BHW, Gehrman PR, Ramautar J, Mazzotti DR, et al. Sleep classification from wrist-worn accelerometer data using random forests. Sci Rep. 2021;11(1):24. doi: 10.1038/s41598-020-79217-x 33420133 PMC7794504

[52] Lettink A, van Wieringen WN, Altenburg TM, Chinapaw MJM, van Hees VT. Inter-Brand, -Dynamic Range, and -Sampling Rate Comparability of Raw Accelerometer Data as Used in Physical Behavior Research. Journal for the Measurement of Physical Behaviour. 2024;7(1).

[53] Skovgaard EL, Roswall MA, Pedersen NH, Larsen KT, Grøntved A, Brønd JC. Generalizability and performance of methods to detect non-wear with free-living accelerometer recordings. Sci Rep. 2023;13(1):2496. doi: 10.1038/s41598-023-29666-x 36782015 PMC9925815

[54] Analog Devices. ADXL345 Data Sheet. Analog Devices. 2022.

[55] Zhou S-M, Hill RA, Morgan K, Stratton G, Gravenor MB, Bijlsma G, et al. Classification of accelerometer wear and non-wear events in seconds for monitoring free-living physical activity. BMJ Open. 2015;5(5):e007447. doi: 10.1136/bmjopen-2014-007447 25968000 PMC4431141

[56] Rasmussen MGB, Pedersen J, Olesen LG, Brage S, Klakk H, Kristensen PL, et al. Short-term efficacy of reducing screen media use on physical activity, sleep, and physiological stress in families with children aged 4-14: study protocol for the SCREENS randomized controlled trial. BMC Public Health. 2020;20(1):380. doi: 10.1186/s12889-020-8458-6 32293374 PMC7092494

[57] Texas Instruments. TMP1075 Data Sheet. Texas Instruments. 2024.

[58] Pagnamenta S, Grønvik KB, Aminian K, Vereijken B, Paraschiv-Ionescu A. Putting temperature into the equation: development and validation of algorithms to distinguish non-wearing from inactivity and sleep in wearable sensors. Sensors. 2022;22(3):1117. doi: 10.3390/s22031117 PMC8838557 35161862

[59] Syed S, Morseth B, Hopstock LA, Horsch A. A novel algorithm to detect non-wear time from raw accelerometer data using deep convolutional neural networks. Sci Rep. 2021;11(1):8832. doi: 10.1038/s41598-021-87757-z 33893345 PMC8065130

[60] Ahmadi MN, Pavey TG, Trost SG. Machine Learning Models for Classifying Physical Activity in Free-Living Preschool Children. Sensors. 2020;20(16):4364. doi: 10.3390/s20164364 PMC7472058 32764316

[61] van Hees VT, Gorzelniak L, Dean León EC, Eder M, Pias M, Taherian S, et al. Separating movement and gravity components in an acceleration signal and implications for the assessment of human daily physical activity. PLoS One. 2013;8(4):e61691. doi: 10.1371/journal.pone.0061691 23626718 PMC3634007

[62] Barouni A, Ottenbacher J, Schneider J, Feige B, Riemann D, Herlan A. Ambulatory sleep scoring using accelerometers—distinguishing between nonwear and sleep/wake states. PeerJ. 2020;8:e8284. 10.7717/peerj.8284 PMC6942683 31915581

[63] Killick R, Fearnhead P, Eckley IA. Optimal Detection of Changepoints With a Linear Computational Cost. Journal of the American Statistical Association. 2012;107(500):1590–8. doi: 10.1080/01621459.2012.737745

[64] Killick R. Methods for changepoint detection. R package changepoint. https://www.r-project.org 2024.

[65] Langford J, Fawcett T, Price E, Bradley D, Wilson A, Croft D. Bio-logging reveals heritable patterns of natural behaviours in sheep. Acta IMEKO. 2024;13(1):1–6. doi: 10.21014/actaimeko.v13i1.1733

[66] Esliger DW, Rowlands AV, Hurst TL, Catt M, Murray P, Eston RG. Validation of the GENEA Accelerometer. Med Sci Sports Exerc. 2011;43(6):1085–93. doi: 10.1249/MSS.0b013e31820513be 21088628

[67] Gifford RM, Greeves JP, Wardle SL, O’Leary TJ, Double RL, Venables M, et al. Measuring the Exercise Component of Energy Availability during Arduous Training in Women. Med Sci Sports Exerc. 2021;53(4):860–8. doi: 10.1249/MSS.0000000000002527 33017351

[68] Price E, Langford J, Fawcett TW, Wilson AJ, Croft DP. Classifying the posture and activity of ewes and lambs using accelerometers and machine learning on a commercial flock. Applied Animal Behaviour Science. 2022;251:105630. doi: 10.1016/j.applanim.2022.105630

[69] Rowlands AV, Powell SM, Humphries R, Eston RG. The effect of accelerometer epoch on physical activity output measures. Journal of Exercise Science & Fitness. 2006;4(1).

[70] Fabre N, Lhuisset L, Bernal C, Bois J. Effect of epoch length on intensity classification and on accuracy of measurement under controlled conditions on treadmill: Towards a better understanding of accelerometer measurement. PLoS One. 2020;15(1):e0227740. doi: 10.1371/journal.pone.0227740 31978093 PMC6980633

[71] Lin LI. A concordance correlation coefficient to evaluate reproducibility. Biometrics. 1989;45(1):255–68. doi: 10.2307/2532051 2720055

[72] Lin LI. A note on the concordance correlation coefficient. Biometrics. 2000;56(1):324–5.

[73] Fraysse F, Post D, Eston R, Kasai D, Rowlands AV, Parfitt G. Physical Activity Intensity Cut-Points for Wrist-Worn GENEActiv in Older Adults. Front Sports Act Living. 2021;2:579278. doi: 10.3389/fspor.2020.579278 33521631 PMC7843957

[74] Boddy LM, Noonan RJ, Rowlands AV, Hurter L, Knowles ZR, Fairclough SJ. The backwards comparability of wrist worn GENEActiv and waist worn ActiGraph accelerometer estimates of sedentary time in children. J Sci Med Sport. 2019;22(7):814–20. doi: 10.1016/j.jsams.2019.02.001 30803818

[75] Matthews CE, Saint-Maurice P, Freeman JR, Hayes HA, Shreves AH, Doherty A, et al. Performance evaluation of algorithms to estimate daily sedentary time using wrist-worn sensors in free-living adults. J Meas Phys Behav. 2025;8(1):jmpb.2024-0051. doi: 10.1123/jmpb.2024-0051 40838174 PMC12363551

[76] Sanders T, Cliff DP, Lonsdale C. Measuring adolescent boys’ physical activity: bout length and the influence of accelerometer epoch length. PLoS One. 2014;9(3):e92040. doi: 10.1371/journal.pone.0092040 24643129 PMC3958416

[77] Skinner AM, Rowlands AV, Vlachopoulos D, Barker AR, Janz KF, Moore SA. The Influence of Accelerometer Epoch Length on Associations of Physical Activity Intensity and Volume with Bone Outcomes. J Sports Sci Med. 2023;22(1):117–32. doi: 10.52082/jssm.2023.117 36876186 PMC9982530

[78] World Health Organization. Measuring physical activity in adults using wearable technologies: report of a scientific meeting. Geneva: WHO. 2025.

[79] Beauchamp MK, D’Amore C, Raina P, McIlroy W, Adesina N, Ahmadi M, et al. Establishing global standards on wearable technology for measuring mobility in ageing populations: an international consensus exercise. Age Ageing. 2026;55(1):afaf376. doi: 10.1093/ageing/afaf376 41563342 PMC12821365

