# Supplementary material for: Validation and optimisation of wearable accelerometer data pre-processing for digital measure implementation and development
Source: PLoS One. 2026 Oct 21;21(10):e0346458. doi: 10.1371/journal.pone.0346458 (PMC13686512; doi:10.1371/journal.pone.0346458)
Supplement: Supplementary file 1 [file pone.0346458.s001.pdf]

S1 Table. Non-wear detection methods.

Authors	Method description				
	Features	Minimum detection	Resolution	Criterion measure	Wear location
van Hees et al. 2011 [41]	Consecutive epochs of 30 minutes are used to calculate the mean and range of each of the acceleration axes. An epoch is classified as non-wear if the standard deviation is less than 3m*g* or range is less than 50m*g*, for at least two out of three axes.				
	Acceleration SD & range	30 minutes	30 minutes	-	Wrist
van Hees et al. 2013 [56]	Epochs of 60 minutes with a 75% overlap are classified as non-wear if the standard deviation is less than 3m*g* or range is less than 50m*g*, for at least two out of three axes. Bordering rules are iteratively applied to non-wear periods of 3 hours and 6 hours based on post and prior non-wear.				
	Acceleration SD & range	60 minutes	15 minutes	-	Wrist
da Silva et al. 2014 [58]	Epochs of 60 minutes with a 75% overlap are classified as non-wear if the standard deviation is less than 13m*g* or range is less than 50m*g*, for at least two out of three axes.				
	Acceleration SD & range	60 minutes	15 minutes	-	Wrist
Zhou et al. 2015 [49]	Epochs of 1 minute incremented by 1 second are classified as non-wear if the temperature is below 26°C and acceleration standard deviation is less than 13m*g* in all three axes.				
	Acceleration SD, absolute temperature	15 minutes	1 minute	Participant diary	Wrist
Ahmadi et al. 2020 [44]	Epochs of 30 minutes incremented by 1 second are classified as non-wear using different rules: standard deviation less than 13m*g*, high-pass filtered sum vector magnitude greater than zero and posture change. Bordering rules are applied to non-wear periods of 30 minutes or less.				
	Acceleration SD, high-pass filtered SVM, posture changes	10 minutes	30 seconds	Observer diary	Wrist

Rasmussen et al. 2020 [50]	Epochs of 10s where the standard deviation of acceleration is below 20m*g* are combined and classified as non-wear if they are longer than 120 minutes, or 45-120 minutes with an average temperature below a threshold, or 10-45 minutes with an average temperature below a threshold and an end time in the expected wake time.				
	Acceleration SD, time of day, temperature	10 minutes	10 seconds	-	Hip & thigh
Barouni et al. 2020 [57]	Epochs of 10 minutes incremented by 30 seconds are classified as non-wear if the power of movement frequencies between 0.1Hz and 0.4Hz is less than 2×10^{-5}. Periods of continuous non-wear are checked to ensure they meet the above criteria at least 75% of the time.				
	Acceleration spectral power	10 minutes	30 seconds	Observer diary & expert rating	Wrist & chest
Rahimi-Eichi et al. 2021 [42]	Epochs of 1 minute are used in a rolling 150-minute window. The standard deviation of each axis in each epoch is calculated. Periods are defined as non-wear when the root mean square of the moving average is below 18.5m*g* for all axes.				
	Acceleration SD	~ 150 minutes	1 minute	Participant diary	Wrist
Sundararajan et al. 2021 [45]	Epochs of 30s are used to calculate features of posture, posture change, mean acceleration and 30 minutes smooth activity score. A random forest machine approach is used to determine non-wear with posture, posture change in bordering epochs and activity scores being the most important features.				
	Posture, posture changes, activity score	5 minutes	30 seconds	Participant diary & expert rating	Wrist
Syed et al. 2021 [54]	Candidate non-wear events are created from epochs of 1 minute incremented by 1 second where the standard deviation of acceleration is below 4m*g*. Bordering non-wear events within 5 minutes of each other are merged. Raw acceleration data for the 3 second periods bordering the candidate non-wear event are processed by a convolutional neural network to detect wear start and wear end.				
	Raw acceleration in a convolutional neural network	1 minute	1 second	Separate wearable device	Hip
Pagnamenta et al. 2022 [52]	Events starting and ending with a temperature change of 3°C over 5 minutes are classified as non-wear if the standard deviation of the band-pass filtered sum vector magnitude is less than 13m*g*.				
	Acceleration SD, rate of change of temperature	5 minutes	1 minute	Participant diary	Lower back

Vert et al. 2022 [43]	Epochs of 5 minutes incremented by 1 minute using a low-pass filtered temperature signal and a 4-second rolling standard deviation of acceleration. Non-wear starts when the 1-minute standard deviation of acceleration is less than 8m*g* for 2 or more axes, standard deviation of acceleration is less than 8m*g* for 2 or more axes 90% of the time over 5 minutes, temperature is dropping more than 0.2 °C per minute and temperature is below 26 °C. Non-wear ends when the 1-minute standard deviation of acceleration is greater than 8m*g* for all axes, standard deviation of acceleration is greater than 8m*g* for 2 or more axes for 50% of the time over 5 minutes, temperature is rising more than 0.1 °C per minute and temperature is above 26 °C.				
	Acceleration SD, temperature, rate of change of temperature	5 minutes	1 minute	Expert rating	Wrist
Skovgaard et al. 2023 [47]	Epochs of 10 seconds are used to calculate features of standard deviation of acceleration, mean acceleration, temperature and time of day. These are then processed by a machine learning decision tree to classify non-wear.				
	Acceleration SD & mean, temperature, time of day	10 seconds	10 seconds	Expert rating	Hip, thigh & wrist
Barakat et al. 2024 [53]	1-minute epoch summaries of acceleration and temperatures are used to create a rolling 3-minute average temperature change and rolling 2-minute averages of acceleration standard deviation. Non-wear events are initiated by a concurrent −0.2°C drop in average temperature and an average acceleration standard deviation below 13 m*g* across all 3 axes. Non-wear events are terminated by an average temperature increase of 0.1 °C accompanied by an increase of the acceleration standard deviation of any of the three axes above 13 m*g*. Only validated during day wear.				
	Acceleration SD, rate of change of temperature	3 minutes	2 minutes	Participant diary	Arm sling

Descriptions of different methods for the detection of non-wear including: the general method, features used for detection, the minimum detectable non-wear duration, the time resolution of non-wear periods, the criterion used in their validation and the wear location.
