# Supplementary material for: Validation and optimisation of wearable accelerometer data pre-processing for digital measure implementation and development
Source: PLoS One. 2026 Oct 21;21(10):e0346458. doi: 10.1371/journal.pone.0346458 (PMC13686512; doi:10.1371/journal.pone.0346458)
Supplement: Supplementary file 2 [file pone.0346458.s002.pdf]

S2 Table. Measurement period information.

<table>
<tr><th>Data object name</th><th>Format</th><th>Technical description</th></tr>
<tr><td>file_history</td><td>array</td><td>Timestamped log of GENEAcore processes completed during a run of the pre-processing pipeline.</td></tr>
<tr><td>errors</td><td>array</td><td>Log of any errors produced during a run of the pre-processing pipeline.</td></tr>
<tr><td>GENEAcore_version</td><td>string</td><td>Version number of the GENEAcore package used to generate the measurement period information (MPI).</td></tr>
<tr><td>line_numbers</td><td>array</td><td>Line numbers of bin file content as a pointer for indexing of bin file sections.</td></tr>
<tr><td>measurement_numbers</td><td>array</td><td>Index of measurements, offset from the first measurement of the file, of the first second, minute, hour, UTC day and local day.</td></tr>
<tr><td>file_info</td><td>array</td><td>Summary of the bin file data size (e.g., number of measurements and page count) and format (e.g., decimal separator).</td></tr>
<tr><td>file_data</td><td>array</td><td>Summary of meta data derived from the bin file header and pre-processing - containing device information, configuration information and study information, e.g.:
<table>
<tr><td>UniqueBinFileIdentifier</td><td>The unique identifier for the bin file.</td></tr>
<tr><td>BinfileName</td><td>The file name of the bin file from which the data was extracted.</td></tr>
<tr><td>ClockDrift</td><td>The clock drift on the device over the measurement period.</td></tr>
<tr><td>ConfigTimeISO</td><td>The local device configuration time, reported in ISO format.</td></tr>
<tr><td>ExtractTimeISO</td><td>The local device extraction time, reported in ISO format.</td></tr>
<tr><td>MeasurementDeviceFirmwareVersion</td><td>The firmware version of the measurement device.</td></tr>
<tr><td>MeasurementDeviceID</td><td>The serial number of the measurement device.</td></tr>
</table>
</td></tr>
</table>

		MeasurementDurationActual	The measurement duration set at configuration.
		MeasurementFrequency	The sampling frequency of the recording.
		MeasurementStartTimeISO	The local recording start time, reported in ISO format.
		MeasurementEndTimeISO	The local recording end time, reported in ISO format.
		SiteID	The unique identifier of the study site.
		StudyID	The unique identifier of the study.
		PeriodInfo	The unique identifier of the study visit.
		ParticipantID	The unique identifier of the study participant.
		TimeZone	The time zone of the device set at configuration.
		VoltsEnd	The battery voltage recorded on the last page of the recording.
		VoltsStart	The battery voltage recorded on the first page of the recording.
		WearLocationConfig	The location of device wear set at configuration.
		NumberDays	The number of days of recorded data.
factory_calibration	array	Calibration values for x, y, z, and light configured during manufacturing.	
auto_calibration	array	Calibration values for x, y, and z derived from recorded data.	
still_bouts	s	Start time and duration of device non-movement events that do not meet the non-wear criteria.	
non_wear	s	Start time and duration of device non-movement events that meet the non-wear criteria.	
button	array	Unique whole second timestamps of event marker occurrences.	

The data structure of the original meta-data from the recording, file management parameters and record of all pre-processing steps.
