# Supplementary material for: Validation and optimisation of wearable accelerometer data pre-processing for digital measure implementation and development
Source: PLoS One. 2026 Oct 21;21(10):e0346458. doi: 10.1371/journal.pone.0346458 (PMC13686512; doi:10.1371/journal.pone.0346458)
Supplement: Supplementary file 3 [file pone.0346458.s003.pdf]

S3 Table. Processing parameters.

Identifier	Units	Value	Technical description	References
still_seconds	s	120	The number of seconds included in the rolling standard deviation calculation of non-movement from 1Hz downsampled data.	Barakat et al. 2024 [43]
sd_threshold	*g*	0.013	The threshold applied to the rolling standard deviation of mean acceleration standard deviation of 1Hz downsampled data to determine non-movement.	da Silva et al. 2014 [44] with formal validation in this work.
spherecrit	*g*	0.3	The minimum required acceleration value for each axis in both directions to ensure sufficient range of non-movement positions for auto-calibration to be reliable.	van Hees et al. 2014 [45]
maxiter	count	500	The maximum number of sphere fit iterations attempted during auto-calibration to converge.	Originally 1000 in van Hees et al. 2014 [45] and optimised in this work.
tol	*g*	1×10^{-13}	The limit of incremental sphere fit improvements before auto-calibration is considered complete.	Originally 1×10^{-12} in van Hees et al. 2014 [45] and optimised in this work.
temp_seconds	s	240	The number of seconds included in the rolling temperature difference calculation for non-wear, which also determines the shortest detection duration.	Vert et al. 2022 [49], Pagnamenta et al. 2022 [58], Barakat et al. 2024 [43]
delta_temp_threshold	°C	-0.7	The threshold applied to the rolling temperature difference to determine non-wear.	Vert et al. 2022 [49], Pagnamenta et al. 2022 [58], Barakat et al. 2024 [43]
x_cpt_penalty	-	18	The manual penalty value applied in the PELT changepoint algorithm for the x axis.	Defined and formally validated in this work.
y_cpt_penalty	-	25	The manual penalty value applied in the PELT changepoint algorithm for the y axis.	Defined and formally validated in this work.
z_cpt_penalty	-	16	The manual penalty value applied in the PELT changepoint algorithm for the z axis.	Defined and formally validated in this work.
minimum_event_duration	s	5	The minimum interval between changepoint transitions.	Twaites et al. 2020 [28] and optimised in this work.
border_seconds	s	300	The maximum number of seconds between non-movement events for them to be combined into the same period.	van Hees et al. 2011 [47], Ahmadi et al. 2020 [50] and optimised in this work.

long_still_seconds	s	7200	The number of seconds for any single non-movement event beyond which the whole period is classed as non-wear.	Rahimi-Eichi et al. 2021 [48] and optimised in this work.
posture_changes_max	count	2	The maximum number of adjoining non-movement events that make up a single period of non-wear less than the maximum non-move duration.	Ahmadi et al. 2020 [50], Sundararajan et al. 2021 [51] and optimised in this work.
non_move_duration_max	s	43200	The number of seconds beyond which non-movement periods are automatically classed as non-wear.	Slovgaard et al. 2023 [53]

The definitions, values and the supporting references for the parameters used in the pre-processing operations.
