# Supplementary material for: Validation and optimisation of wearable accelerometer data pre-processing for digital measure implementation and development
Source: PLoS One. 2026 Oct 21;21(10):e0346458. doi: 10.1371/journal.pone.0346458 (PMC13686512; doi:10.1371/journal.pone.0346458)
Supplement: Supplementary file 4 [file pone.0346458.s004.pdf]

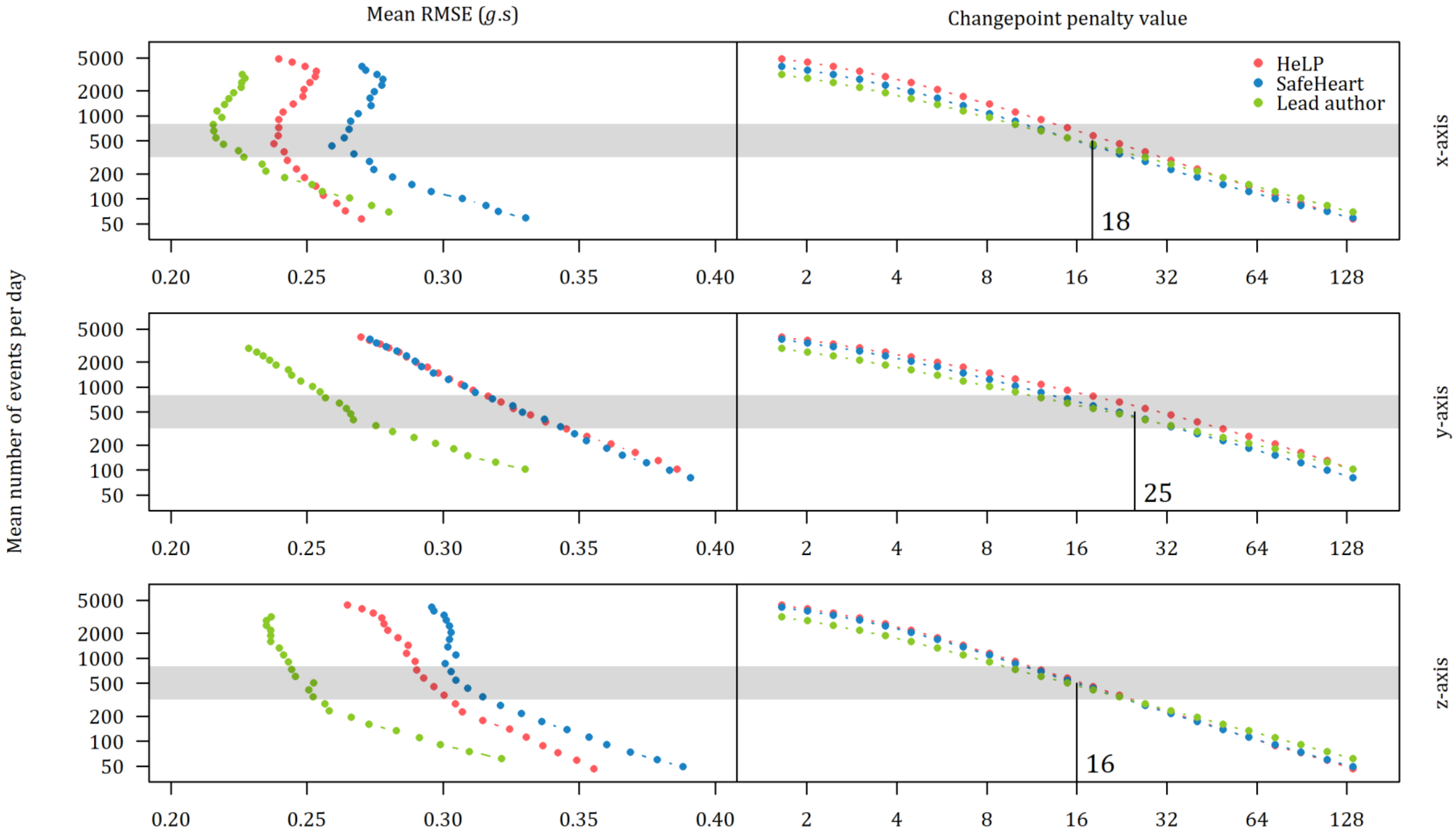

S1 Fig. Changepoint penalty value assessments. The variation of the number of daily events and the RMSE of intensity with changepoint penalty value for each axis of acceleration in the 3 verification population groups.
