# Supplementary material for: Validation and optimisation of wearable accelerometer data pre-processing for digital measure implementation and development
Source: PLoS One. 2026 Oct 21;21(10):e0346458. doi: 10.1371/journal.pone.0346458 (PMC13686512; doi:10.1371/journal.pone.0346458)
Supplement: Supplementary file 5 [file pone.0346458.s005.pdf]

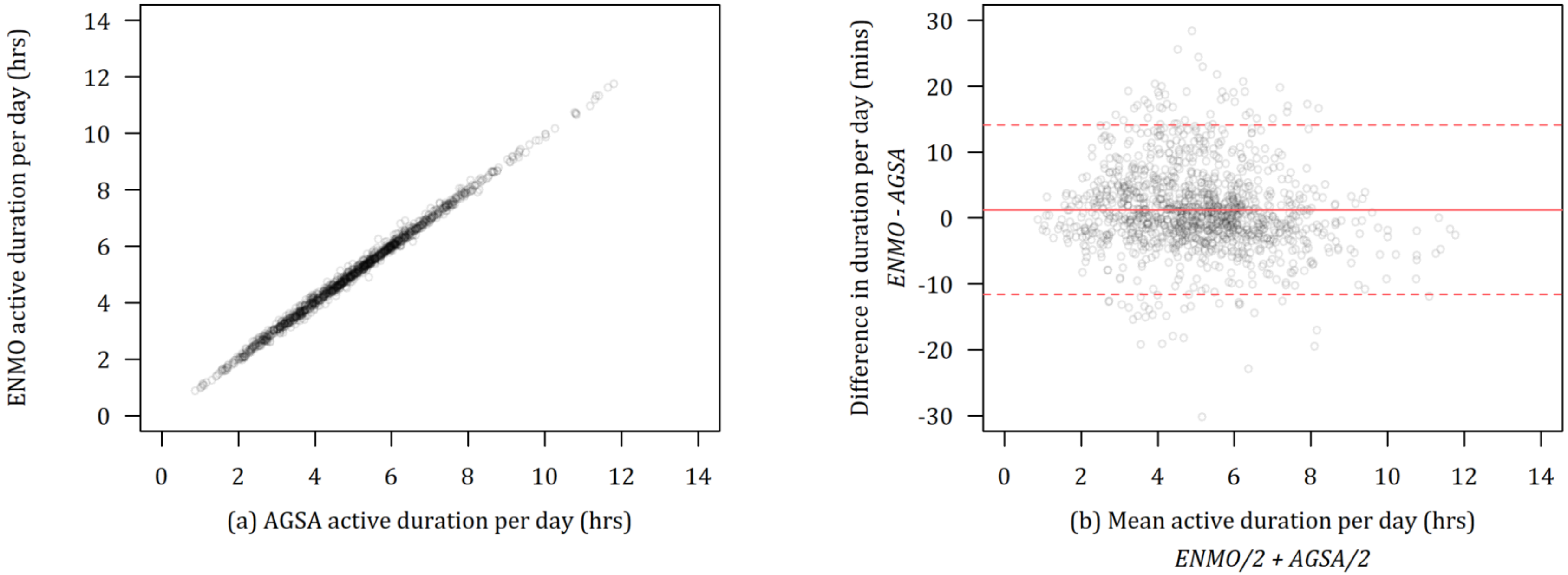

S2 Fig. Comparing daily active duration using AGSA and ENMO. (a) Correlation plot of active hours per day as assessed by AGSA and ENMO, (b) Bland-Altman plot of the means and differences of AGSA and ENMO active hours per day with mean bias and 95% SD confidence intervals.
