# Supplementary material for: Validation and optimisation of wearable accelerometer data pre-processing for digital measure implementation and development
Source: PLoS One. 2026 Oct 21;21(10):e0346458. doi: 10.1371/journal.pone.0346458 (PMC13686512; doi:10.1371/journal.pone.0346458)
Supplement: Supplementary file 6 [file pone.0346458.s006.pdf]

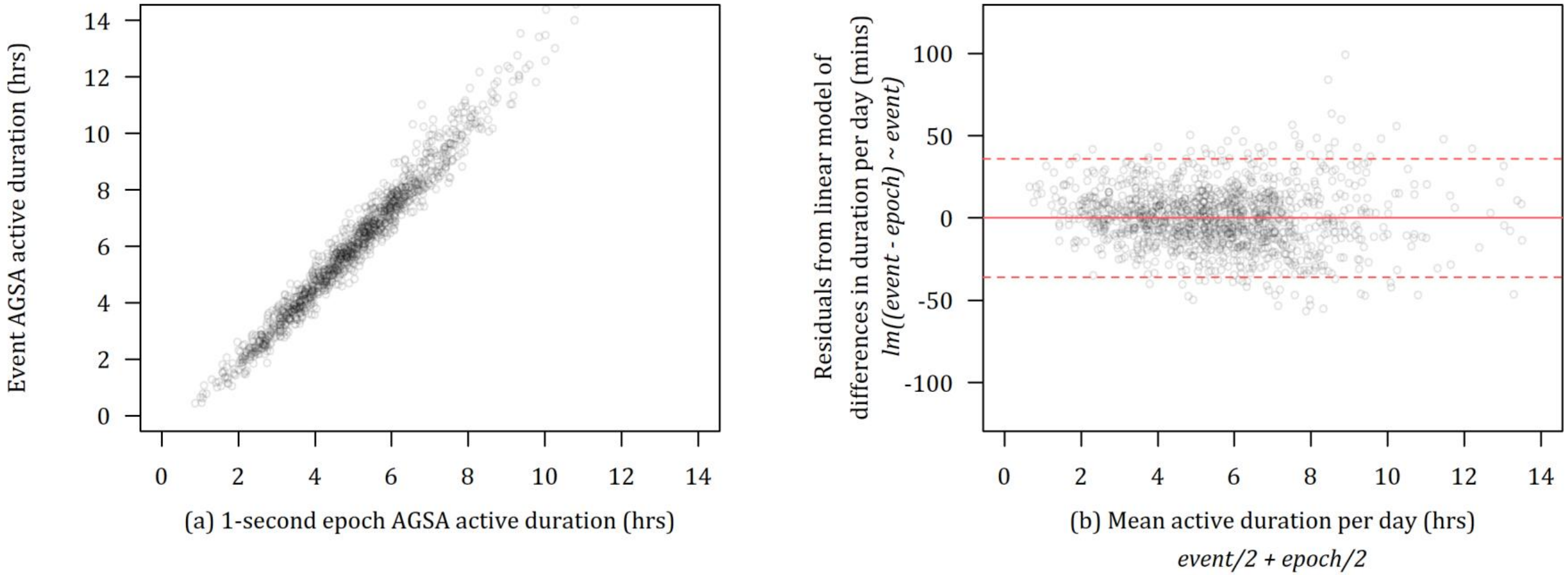

S3 Fig. Comparing daily active duration using event and epoch aggregates of AGSA. (a) Correlation plot of active hours per day as assessed by AGSA 1s epoch and variable duration events, (b) Bland-Altman regression plot modelling the differences of AGSA 1s epoch and variable duration event active hours by event-assessed duration per day with mean bias and 95% SEE confidence intervals.
